# Artificial small RNA for sequence specific cleavage of target RNA through RNase III endonuclease Dicer

**DOI:** 10.18632/oncotarget.9582

**Published:** 2016-05-25

**Authors:** Wen Xu, Yuchen Liu, Yali Liu, Li Liu, Yonghao Zhan, Chengle Zhuang, Junhao Lin, Mingwei Chen, Jianfa Li, Zhiming Cai, Weiren Huang, Yong Zhang

**Affiliations:** ^1^ State Key Laboratory of Biocontrol, Institute of Aquatic Economic Animals, and the Guangdong Province Key Laboratory for Aquatic Economic Animals, School of Life Sciences, Sun Yat-Sen University, Guangzhou 510275, China; ^2^ Key Laboratory of Medical Reprogramming Technology, Shenzhen Second People's Hospital, The First Affiliated Hospital of Shenzhen University Shenzhen, Shenzhen 518000, China; ^3^ Shantou University Medical College, Shantou 515041, China; ^4^ South China Sea Bio-Resource Exploitation and Utilization Collaborative Innovation Center, Guangzhou 510275, China

**Keywords:** artificial RNA, dicer, gene regulation

## Abstract

CRISPR-Cas9 system uses a guide RNA which functions in conjunction with Cas9 proteins to target a DNA and cleaves double-strand DNA. This phenomenon raises a question whether an artificial small RNA (asRNA), composed of a Dicer–binding RNA element and an antisense RNA, could also be used to induce Dicer to process and degrade a specific RNA. If so, we could develop a new method which is named DICERi for gene silencing or RNA editing. To prove the feasibility of asRNA, we selected MALAT-1 as target and used Hela and MDA-MB-231 cells as experimental models. The results of qRT-PCR showed that the introduction of asRNA decreased the relative expression level of target gene significantly. Next, we analyzed cell proliferation using CCK-8 and EdU staining assays, and then cell migration using wound scratch and Transwell invasion assays. We found that cell proliferation and cell migration were both suppressed remarkably after asRNA was expressed in Hela and MDA-MB-231 cells. Cell apoptosis was also detected through Hoechst staining and ELISA assays and the data indicated that he numbers of apoptotic cell in experimental groups significantly increased compared with negative controls. In order to prove that the gene silencing effects were caused by Dicer, we co-transfected shRNA silencing Dicer and asRNA. The relative expression levels of Dicer and MALAT-1 were both detected and the results indicated that when the cleavage role of Dicer was silenced, the relative expression level of MALAT-1 was not affected after the introduction of asRNA. All the above results demonstrated that these devices directed by Dicer effectively excised target RNA and repressed the target genes, thus causing phenotypic changes. Our works adds a new dimension to gene regulating technologies and may have broad applications in construction of gene circuits.

## INTRODUCTION

As adaptive immune defenses of bacteria and archaea, clustered regularly interspaced short palindromic repeats (CRISPR)/CRISPR-associated (Cas) systems have become a general and powerful tool for genome editing, especially the type II bacterial CRISPR/Cas9 system [1–4]. CRISPR loci are composed of a series of repeats, which are separated by “spacer” sequences. The “spacer” sequences match the genomes of bacteriophages and other mobile genetics elements [5–7]. The repeat spacer array is transcribed and processed to generate a small crRNA to recognize the target sequences [8–12]. The element flanking the repeat spacer array is the CRISPR-associated (cas) gene encoding the Cas9, a double-stranded DNA endonuclease that employs the crRNA to guide the cleavage of target site [13]. A sequence motif in the downstream of the target site, known as protospacer-adjacent motif (PAM), is essential to the cleavage [14–15]. And the loading of the crRNA onto Cas9 also requires a small tracrRNA which is antisense to the crRNA precursor and RNase III [14]. Now scientists have successfully fused the crRNA and tracrRNA to generate a small guide RNA to simplify the system [16].

RNA interference (RNAi) has challenged our view of mechanisms regulating the expression of genetic information [17–18]. It indicates that not only proteins but RNA molecules could regulate gene expression in Eukaryotes [19]. Firstly, in the nucleus, a 50–70nt stem-loop precursor (pre-miRNA) is excised from a primary transcript (pri-miRNA) by Drosha [20–21]. And then the nuclear transport receptor, exportin-5, transports the pre-miRNA to the cytoplasm. Subsequently, the pre-miRNA is cleaved by Dicer [22], generating a short 19–23nt duplex with 2nt overhangs at the 3′-ends and phosphorylated 5′-termini. After the short duplex is loaded onto a multicomponent nuclease RISC, one strand is released and degraded, and the other remains severing as a guide sequence to instruct RISC to destroy the complementary messenger RNA (mRNA) [23–25]. Inspired by the role model of CRISPR/Cas9 systems, we asked whether we could introduce an artificial small RNA (asRNA), composed of a Dicer–binding RNA element and an antisense RNA, to induce Dicer to process and degrade a specific RNA, just like using the guide RNA to induce Cas9 to cleave the target sequences in the CRISPR/Cas9 systems. We named our new method as “DICERi” in this study, and we verified our hypothesis from different aspects.

## RESULTS

### Design and construction of the asRNA expression plasmid vectors

**Figure 1:**
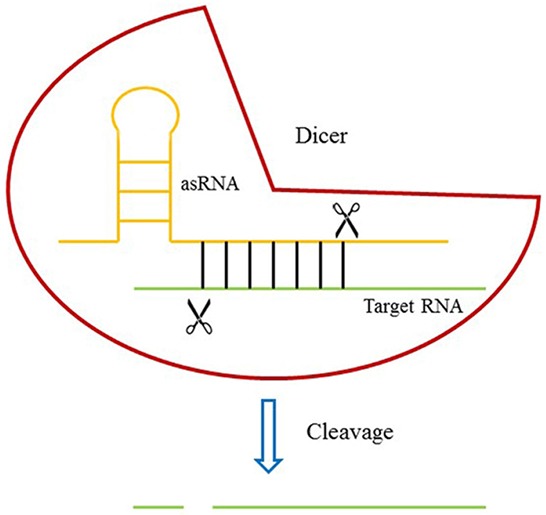
The design of DICERi Dicer protein binds to target RNA through an asRNA and induces the cleavage of target RNA.

We used an oligomer RNA [26] (Supplementary Table S1) which had a great affinity for Dicer (but not cleaved by Dicer) and added it to an antisense RNA targeting MALAT-1 RNA to form an artificial small RNA (asRNA) (Supplementary Table S2) (Figure 1). The plasmid pGPU6-GFP-NEO was used to express the asRNA after the cDNA sequence of asRNA was inserted into it. The plasmid vector which could only express the oligomer was also constructed and used as negative control.

### The effects of asRNA on the expression level of target gene in Hela cell and MDA-MB-231 cell

**Figure 2:**
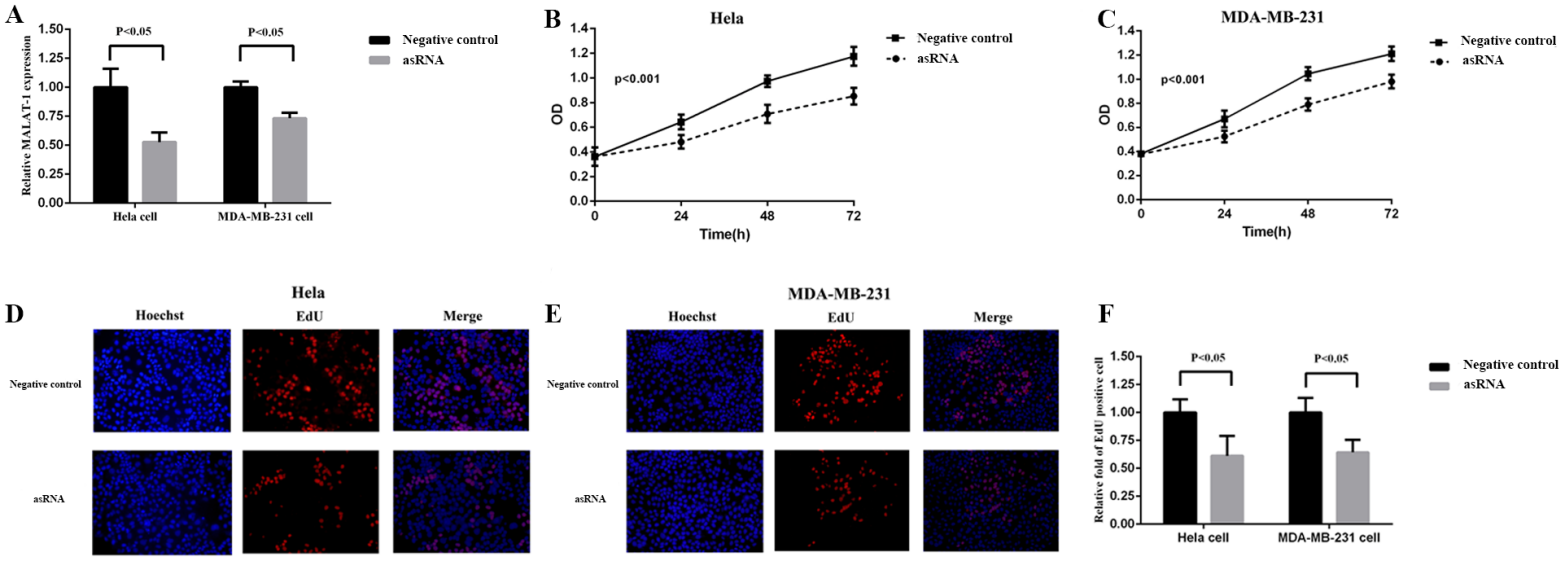
Effects of transfection of MALAT-1 asRNA vector on relative MALAT-1 expression level and cell proliferation in Hela cell and MDA-MB-231 cell **A.** The relative expression level of MALAT-1 was evaluated using qRT-PCR. The MALAT-1 asRNA significantly down-regulated the expression level of MALAT-1 in Hela cell and MDA-MB-231cell (p<0.05). **B, C.** Cell proliferation was detected by CCK-8 assay at 0, 24, 48, 72h. ANOVA was used to analyze the curves of cell proliferation. asRNA inhibited the cell proliferation of Hela cell and MDA-MB-231 cell (P<0.001). **D, E, F.** EdU staining assay was used to detect cell proliferation. Hoechst reagent stains all cell nuclei and EdU reagent only stains the newly-proliferated cell nuclei. The calculation of relative fold of EdU positive cell showed that asRNA dramaticlly suppressed cell proliferation both in Hela and MDA-MB-231 cell (p<0.05). Data are shown as mean±SD. The experiments were performed in triplicate for three independent times.

We chose metastasis-associated lung adenocarcinoma transcript 1 (MALAT-1) as a target gene, and transfected the plasmids expressing the corresponding asRNA or oligomer RNA into Hela and MDA-MB-231 cells. We examined the expression level of MALAT-1 using qRT-PCR at 48h post-transfection. As the results showed, the asRNA induced a significant decrease in the expression level of MALAT-1 compared with the negative controls (Figure 2A).

### The effects of asRNA on cell proliferation in Hela cell and MDA-MB-231 cell

MALAT-1, a long non-coding RNA, is involved in cell proliferation in cancer cells, so we next measured the cell proliferation after the transfections of plasmids expressing the corresponding asRNA or oligomer RNA into Hela cell and MDA-MB-231 cell. The results of CCK-8 (Figure 2B, 2C) and EdU (Figure 2D, 2E, 2F) assays both indicated that cell proliferation was suppressed remarkably after asRNA was expressed in Hela and MDA-MB-231 cells.

### The effects of asRNA on cell migration in Hela cell and MDA-MB-231 cell

**Figure 3:**
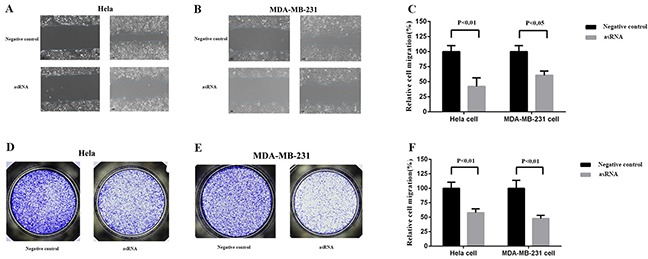
Effect of transfection of MALAT-1 asRNA vector on cell migration in Hela cell and MDA-MB-231 cell Cell migration was analyzed using wound scratch assay **A, B, C.** and Transwell assay **E, F, G.** Percentage of relative cell migration was calculated to reflect the cell number of migration. Compared with negative control groups, the cell migration was remarkably repressed in the experiment groups in both Hela cell (wound scratch assay, p<0.01; Transwell assay, p<0.05) and MDA-MB-231 cell (wound scratch assay, p<0.01; Transwell, p<0.01). Data are shown as mean±SD. The experiments were performed in triplicate for three independent times.

MALAT-1 is also associated with cell migration in cancer cells, we next tested the effects of asRNA on cell migration in Hela cell and MDA-MB-231 cell. Wound scratch assay (Figure 3A, 3B, 3C) and Transwell invasion assay (Figure 3D, 3E, 3F) were employed after the transfections of plasmids expressing the corresponding asRNA or oligomer RNA into Hela cell and MDA-MB-231 cell. The results of both assays suggested that the asRNAs could effectively repress cell migration.

### The effects of asRNA on cell apoptosis in Hela and MDA-MB-231 cells

**Figure 4:**
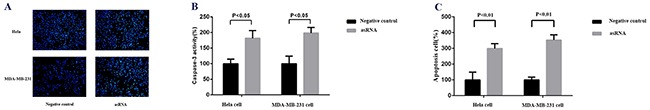
Effect of transfection of MALAT-1 asRNA vector on cell apoptosis in Hela cell and MDA-MB-231 cell **A, B.** Cell apoptosis was detected by Hoechst staining assay. Hoechst reagent stains the apoptotic cells more brightly than normal cells. The percentage of caspase-3 activity was calculated to reflect the cell apoptosis and showed that MALAT-1 asRNA suppressed cell apoptosis significantly in Hela cell and MDA-MB-231 cell (p<0.05 in each cell line). **C.** ELISA assay was used to detect the cell apoptosis. At 48 post transfections of corresponding vectors, cells were digested, lysed and then handled using ELISA kit. Percentage of apoptotic cell was calculated to reflect the cell apoptosis and the results suggested that MALAT-1 asRNA suppressed cell apoptosis significantly in Hela cell and MDA-MB-231 cell (p<0.01 in each cell line). Data are shown as mean±SD. The experiments were performed in triplicate for three independent times.

MALAT-1 is also reported to have some relationship with cell apoptosis in cancer cells, so, here we detected the effects of asRNA on cell apoptosis in Hela cell and MDA-MB-231 cell. The results of Hoechst staining (Figure 4A, 4B) and ELISA (Figure 4C) assays demonstrated that the cell apoptosises were inhibited in both types of cells after the transfections of plasmids expressing the asRNA in Hela and MDA-MB-231 cells.

### The effect of asRNA is dependent on Dicer

**Figure 5:**
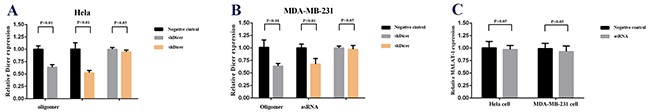
Effect of co-transfection of MALAT-1 asRNA vector and shDicer vector or negative control vector and shDicer vector on relative MALAT-1 expression level in Hela cell and MDA-MB-231 cell **A, B.** qRT-PCR was used to analyzed the relative Dicer expression level in Hela cell and MDA-MB-231 cell. The results showed the shDicer reduced the relative Dicer expression level in Hela cells and MDA-MB-231 cell significantly (p<0.01 in each cell line). **C.** The relative MALAT-1 expression level were detected after co-transfection of MALAT-1 asRNA vector and shDicer vector or negative control vector and shDicer vector in Hela cell and MDA-MB-231 cell using qPCR. The data demonstrated that the relative MALAT-1 expression levels were not affected dramatically when Dicer was silenced in Hela cell and MDA-MB-231 cell (p>0.05 in each line). Data are shown as mean±SD. The experiments were performed in triplicate for three independent times.

To make sure that the repression effects of asRNA were triggered by Dicer, the shRNA targeting Dicer (Supplementary Table 3) and asRNA targeting MALAT-1 were co-transfected into Hela and MDA-MB-231 cells. In negative control groups, shRNA targeting Dicer and oligomer RNA were co-transfected into cells. As expected, when the cleavage role of Dicer was silenced, the repression of target gene was released and the relative expression level of MALAT-1 was not affected (Figure 5).

## DISCUSSION

In this study, we proposed a new method that used an oligomer RNA as a protein-binding RNA element to induce Dicer to degrade target RNA. Firstly, we chose MALAT-1 as target gene and constructed an asRNA (composed of Dicer-binding RNA element and an antisense RNA) expression vector. Then we measured the effects of these devices on the expression level and phenotypic changes, such as cell proliferation, cell migration and cell apoptosis. The results showed that the new method could repress the expression level as well as functions of target gene significantly, compared with the negative controls. Next, we co-transfected shRNA targeting Dicer and asRNA against MALAT-1 to confirm that the suppression was actually induced by Dicer. Whether DNA edition or RNA edition has been widely used for biological and therapeutic purpose. They could specifically activate or suppress DNA or RNA to modulate the gene networks. Here, we repurposed Dicer and added an another member to RNA edition. The results of diverse experiment methods had testified that our hypothesis was correct. However, further work, like improving the specific bind between antisense RNA and target RNA, should be done to make the new method more appliable. In conclusion, our new modus is a novel and effective tool for use of RNA edition and could have a broad application to direct and rewire the gene networks.

## MATERIALS AND METHODS

### Plasmids construction

The plasmid vector pGPU6-GFP-Neo was used to transiently express shRNA and asRNA. The cDNA sequence of shRNA or asRNA was designed, synthesized, and inserted into pGPU6-GFP-Neo vector at restriction site of Bam HI / Bbs I. The relative sequences in the vectors were showed in Supplementary Table 2, and Table 3.

### Cell lines and cell culture

Hela cell and MDA-MB-231 cell used in this study were purchased from the Institute of Cell Research, Chinese Academy of Sciences, Shanghai, China. Hela and MDA-MB-231 cells were maintained in DMEM medium supplemented with 10% fetal bovine serum (Invitrogen, USA) in the presence of 5% CO_2_ at 37°C.

### Cell transfection

For transient transfection experiments, we used Lipofectamine 2000 Transfection Reagent (Invitrogen, USA) to transfect the plasmids according to the manufacturer's instructions.

### RNA extraction and qRT-PCR

Total RNA was extracted from celllines at forty-eight hours post-transfection using TRIzol reagent (Invitrogen, USA) according to the suggested instructions. cDNA was synthesized using PrimeScript^TM^RT-PCR kit(Takara, Japan) according to the manufacturer's instructions. Real time quantitative PCR was performed on an ABI PRISM 7000 Fluorescent Quantitative PCR System (Applied Biosystems, USA) using SYBR Premix Ex Taq ^TM^(Tli RNaseH Plus, Japan)according to the related instructions. The expression level was calculated using 2^−ΔΔCt^t method. Experiments were repeated at least three times in duplicates.

### Cell proliferation assay

CCK-8: Absorbance at a wavelength of 450nm was measured one hour later after Cell Counting Kit-8(BeyotimeInst Biotech, China) reagent was added to each well of a 96-well plate. And the absorbances at 24, 48 and 72h post-transfection were also measured using an Multiscan Go (Thermo, USA). Experiments were repeated at least three times in duplicates.

EdU: Incubation with EdU reagent (Ribobio, China) at 48h post-transfection. And then permeabilization buffer was added to cells at 24h post-incubation followed by washing with PBS. After stained with Apollo solution (Ribobio, China) for 30mins, cells were observed using fluorescence microscopy. Experiments were repeated at least three times in duplicates.

### Cell migration assay

Wound scratch assay: A wound scratch field was created before removing the transfection reagent. Cells were washed several times with PBS after the wound scratch was created and pictures was taken to record the “blank field” at 0h and 16h. Software program HMIAS-2000 was used to calculate the cell migration distance. Experiments were repeated at least three times in duplicates.

Transwell invasion assay: Before cells were transplanted into transwells (8μm, Corning, USA), 700μl DMEM medium with 10%FBS was pipetted into wells of 12-well plate. After 24h of transfection, proper number of cells were transferred to transwells, and the serum-free medium was supplemented to 200μl per well. After appropriate time of incubation, the transwells were stained with 0.5% crystal violet solution and photographed using NIKON H600L, the OD_570_ was measured to reflect the cell number of migration. Experiments were repeated at least three times in duplicates.

### Cell apoptosis assay

ELISA: According to Caspase 3 ELISA assay kit (Cusabio, USA) protocol, cells were firstly lysed and then putted 100μl into wells. The wells covered with adhesive strip were incubated for 30mins at 37°C and then aspirated. After washed three times, each well was incubated firstly with 100μl HPR-conjugate for 30mins at 37°C and then 90μl TMB substrate 20 mins at 37°C. Before TMB substrate was added, each well was aspirated and washed three times. 50μl stop solution was pipetted to every well and then tapped gently. Multiscan GO was used to read absorbance at a wavelength of 450nm. Experiments were repeated at least three times in duplicates.

Hoechst staining assay: Cells were incubated with Hoechst stain for 20mins and pictures were taken using fluorescence microscopy after cells were washed with PBS. The cell apoptosis ratio was calculated to analyze the effect of asRNA. Experiments were repeated at least three times in duplicates.

### Statistical analysis

Statistical analysis was performed using Student's t-test or ANOVA and P < 0.05 was considered statistically significant. All statistical tests were conducted using SPSS version 19.0 software (SPSS, Chicago, IL, USA).

## SUPPLEMENTARY TABLES


