# Supplementary material for: Artificial small RNA for sequence specific cleavage of target RNA through RNase III endonuclease Dicer
Source: Oncotarget. 2016 May 25;7(34):54549–54. doi: 10.18632/oncotarget.9582 (PMC5342362; doi:10.18632/oncotarget.9582)
Supplement: Supplementary file 1 [file oncotarget-07-54549-s001.pdf]

SUPPLEMENTARY TABLES

Supplementary Table S1: Oligomer sequences binding Dicer

5’-GGGAGAAUCAUAAGUAGCGGUGUGUGAGUCGUGGUGCCCCAUGUUAACAGUUAGCC-3’

Supplementary Table S2: cDNAs equences of asRNA targeting MALAT-1 in the vector

5’-GGGAGAATCATAAGTAGCGGTGTGTGAGTCGTGGTGCCCCATGTTAACAGTTAGCCATAAATCCCTTTACACCTC-3’

Note: the underlied alphabets are the oligomer sequences

Supplementary Table S3: shRNA-Dicer sequences in the vector

5’-GCTCGAAATCTTACGCAAATA-3’
